# Pro-inflammatory AGE-RAGE signaling is activated during arousal from hibernation in ground squirrel adipose

**DOI:** 10.7717/peerj.4911

**Published:** 2018-06-04

**Authors:** Samantha M. Logan, Kenneth B. Storey

**Affiliations:** Institute of Biochemistry, Departments of Biology and Chemistry, Carleton University, Ottawa, Ontario, Canada

**Keywords:** AGE-RAGE, Inflammation, 13-lined ground squirrel, Damage-associated molecular pattern molecule, Hibernation, Adipose, Torpor-arousal cycle

## Abstract

**Background:**

Inflammation is generally suppressed during hibernation, but select tissues (e.g. lung) have been shown to activate both antioxidant and pro-inflammatory pathways, particularly during arousal from torpor when breathing rates increase and oxidative metabolism fueling the rewarming process produces more reactive oxygen species. Brown and white adipose tissues are now understood to be major hubs for the regulation of immune and inflammatory responses, yet how these potentially damaging processes are regulated by fat tissues during hibernation has hardly been studied. The advanced glycation end-product receptor (RAGE) can induce pro-inflammatory responses when bound by AGEs (which are glycated and oxidized proteins, lipids, or nucleic acids) or damage associated molecular pattern molecules (DAMPs, which are released from dying cells).

**Methods:**

Since gene expression and protein synthesis are largely suppressed during torpor, increases in AGE-RAGE pathway proteins relative to a euthermic control could suggest some role for these pro-inflammatory mediators during hibernation. This study determined how the pro-inflammatory AGE-RAGE signaling pathway is regulated at six major time points of the torpor-arousal cycle in brown and white adipose from a model hibernator, *Ictidomys tridecemlineatus*. Immunoblotting, RT-qPCR, and a competitive ELISA were used to assess the relative gene expression and protein levels of key regulators of the AGE-RAGE pathway during a hibernation bout.

**Results:**

The results of this study revealed that RAGE is upregulated as animals arouse from torpor in both types of fat, but AGE and DAMP levels either remain unchanged or decrease. Downstream of the AGE-RAGE cascade, *nfat5* was more highly expressed during arousal in brown adipose.

**Discussion:**

An increase in RAGE protein levels and elevated mRNA levels of the downstream transcription factor *nfat5* during arousal suggest the pro-inflammatory response is upregulated in adipose tissue of the hibernating ground squirrel. It is unlikely that this cascade is activated by AGEs or DAMPs. This research sheds light on how a fat-but-fit organism with highly regulated metabolism may control the pro-inflammatory AGE-RAGE pathway, a signaling cascade that is often dysregulated in other obese organisms.

## Introduction

The advanced-glycation end product (AGE) and AGE receptor (AGE-RAGE) pathway is emerging as an important signal transduction pathway that influences an immune and oxidative stress response via the activation of mitogen-activated protein kinase (MAPK) and nuclear factor kappa-light-chain-enhancer of activated B cells (NF-κB) pathways (Sparvero et al., 2009). RAGE is normally expressed in a range of tissues including lung, heart, vasculature, brain, liver, embryonic tissue, etc., but becomes overexpressed in areas of localized inflammation (for instance, post-trauma) or in tissues affected by inflammatory disease (like diabetes, cancer, neurodegeneration, etc.) (Brett et al., 1993; Sparvero et al., 2009). This pro-inflammatory pathway can contribute to disease pathogenesis since its activation (via ligand binding to RAGE) often leads to cellular dysfunction and ultimately tissue damage (Ramasamy, Yan & Schmidt, 2011).

During the winter months, hibernating ground squirrels use a range of molecular adaptations to maintain tissue homeostasis until arousal in the spring. These animals regulate tissue viability through antioxidant (Morin et al., 2008; Ni & Storey, 2010; Rouble, Tessier & Storey, 2014) and anti-apoptotic (Rouble et al., 2013; Logan et al., 2016; Logan, Luu & Storey, 2016) pathways to prevent the conditions (e.g., hypoxia, cell death) that could give rise to localized inflammation and subsequent tissue damage (Bouma et al., 2013; Bogren et al., 2014). However, there is evidence that pro-inflammatory pathways could be upregulated upon arousal from hibernation from reports showing increased oxidized proteins, lipid peroxides, and pro-inflammatory cytokines (TNF-alpha, IFN-γ) during arousal from hibernation in ground squirrels and bats (Lee, Choi & Park, 2002; Kurtz & Carey, 2007; Orr et al., 2009). Hibernating ground squirrels are well known for their extraordinary ability to maintain whole-body homeostasis during periods of cell stress, so it is essential to understand how they control inflammation, a process that could result in cerebral ischemia, muscle wasting, and overconsumption of ATP if left unchecked, but does not in ground squirrels (Frerichs et al., 1994; Bogren et al., 2014; Ruf & Geiser, 2015; Tessier & Storey, 2016). Most studies on inflammation in hibernators focus on how anti-inflammatory/protective signaling pathways could be involved in this whole-body homeostasis but few studies have aimed to identify potential pro-inflammatory pathways that could be activated to warrant the increase in anti-inflammatory signaling, in the first place. The regulation of the AGE-RAGE pathway has never been studied in an animal that undergoes metabolic suppression (to the best of our knowledge). Thus, it is essential to assess how the pro-inflammatory AGE-RAGE pathway may be regulated throughout the torpor-arousal cycle in the 13-lined ground squirrel (*Ictidomys tridecemlineatus*, a model hibernator).

Specifically, the brown and white adipose tissues (BAT and WAT, respectively) of 13-lined ground squirrels are of interest because these tissues undergo major physiological changes before and during hibernation that involve managing oxidative stress and inflammation. Hibernator BAT undergoes a 2- to 3-fold increase in weight during the pre-hibernation phase in Arctic ground squirrels (*Spermophilus parryii*) (Boyer & Barnes, 1999) since BAT is essential for the generation of heat during arousals from torpor via non-shivering thermogenesis (Storey & Storey, 2005). The arousal process involves an increased production of reactive oxygen species (ROS) (accompanying increased oxygen intake and elevated oxidative metabolism) which can lead to tissue damage and inflammation. Evidence of increased ROS production during torpor and/or arousal in mammals includes increased lipid peroxidation, HSP70 expression, and NF-κB activity in ground squirrel intestine (Carey, Frank & Seifert, 2000; Carey, Rhoads & Aw, 2003). Adipose tissues from hibernating European ground squirrel (*Spermophilus citellus*) and 13-lined ground squirrels show increases in antioxidant enzymes such as superoxide dismutases 1 and 2, and catalase, possibly to counteract increases in the generation of ROS (Vucetic et al., 2013; Rouble, Tessier & Storey, 2014). WAT also rapidly expands in the summer months before torpor following hyperphagia (a period of intense eating needed to lay down large depots of stored fatty acids) (Sheriff et al., 2013; Schwartz, Hampton & Andrews, 2015). WAT is also an important mediator of the inflammatory response since it can produce both pro- and anti-inflammatory cytokines termed adipokines (Trayhurn & Wood, 2004). Oxidative stress and chronic inflammation in humans promotes the dysregulation of adipokines (e.g., leptin, TNF-α, IL-6, adiponectin, etc.) synthesis and secretion which can ultimately lead to secondary metabolic disorders including insulin resistance, cardiovascular disease, and cancer (Kayser & Verges, 2013; He et al., 2015). By contrast, the 13-lined ground squirrel is “fat but fit”, providing an excellent model of controlled energy metabolism and regulated inflammatory/immune responses. Thus, it is essential to study the AGE-RAGE pathway and the pro-inflammatory pathways it regulates (i.e., MAPK, JNK, NFAT) in a fat-storing hibernator this is capable of reversible insulin resistance and that is resistant to tissue damage brought on by oxidative stress.

RAGE is a receptor for protein ligands including glycated proteins (AGEs), damage associated molecular patterns (DAMPs), and pathogen associated molecular patterns (PAMPs) (not part of the current study). AGEs are formed by the non-enzymatic attachment of sugars to proteins, lipids, or nucleic acids by the Maillard reaction, a complicated process that can yield hundreds of AGE species and has been discussed elsewhere (Ott et al., 2014; Boyer et al., 2015). AGE accumulation is a symptom of inflammatory states including normal aging and metabolic disorders like diabetes where glucose is openly available (Cooper, 2004; Vlassara, Brownlee & Cerami, 1985). Damage-associated molecular pattern (DAMP) molecules are intracellularly derived proteins released into the extracellular space by dead or dying cells, and are RAGE ligands that can invoke immune, pro-inflammatory, and oxidative stress signaling cascades (Rubartelli & Lotze, 2007). DAMPs that bind RAGE include S100 calcium-binding protein B (S100B) and high mobility group box 1 protein (HMGB1), both of which are highly conserved between mammalian species and are expressed in adipose tissue (Buckman et al., 2014; Ghosh et al., 2016). AGEs or DAMPs binding to RAGE, activates ERK1/2, JNK, and p38 signaling pathways induce pro-inflammatory gene expression (Sparvero et al., 2009; Ott et al., 2014). The focus of this study is the MAPK signaling pathway involving ERK1/2 because this pathway was activated during the stress response in BAT and WAT from torpid primates (Biggar et al., 2015). Raf is a serine/threonine MAPK kinase kinase (MAPKKK) which is involved in the phosphorylation of MAPK kinases (MAPKK) like MEK1/2, which phosphorylates MAPKs (ERK1/2) to activate them. Once activated, ERK1/2 can phosphorylate transcription factors from the Ets, NFAT, and Jun families to promote the expression of inflammatory/immune genes (Cowan & Storey, 2003; MacDonald & Storey, 2005a; Lu & Xu, 2006). Experiments on obese human WAT (a good system for studying oxidative stress and chronic inflammation in adipose) showed that Ets1 can directly regulate inflammation in adipocytes by interacting with the TNF-α promoter and promoting TNF-α expression (Zhang et al., 2016). ERK1/2 also regulates the activation of the Rel family of transcription factors, including NF-κB and NFAT1-5 (Ueno et al., 2013). NFATs regulate immune responses and angiogenesis, making them interesting to study in the context of expanded adipose tissue. NFAT5 is expressed in both WAT and BAT, is known to associate with NF-κB to increase its pro-inflammatory activity, and can regulate the expression of important immune and osmoregulatory genes like monocyte chemoattractant protein 1 (MCP1) and TNF-α (Ueno et al., 2013). Finally, Jun, one of the subunits of the AP-1 transcription factor, is an important mediator of inflammation in adipocytes. Elevated leptin levels activate Jun and AP-1 DNA binding to increase reactive oxygen species production and to propagate pro-inflammatory signaling (Gullicksen et al., 2003; Makki, Froguel & Wolowczuk, 2013).

**Figure 1:**
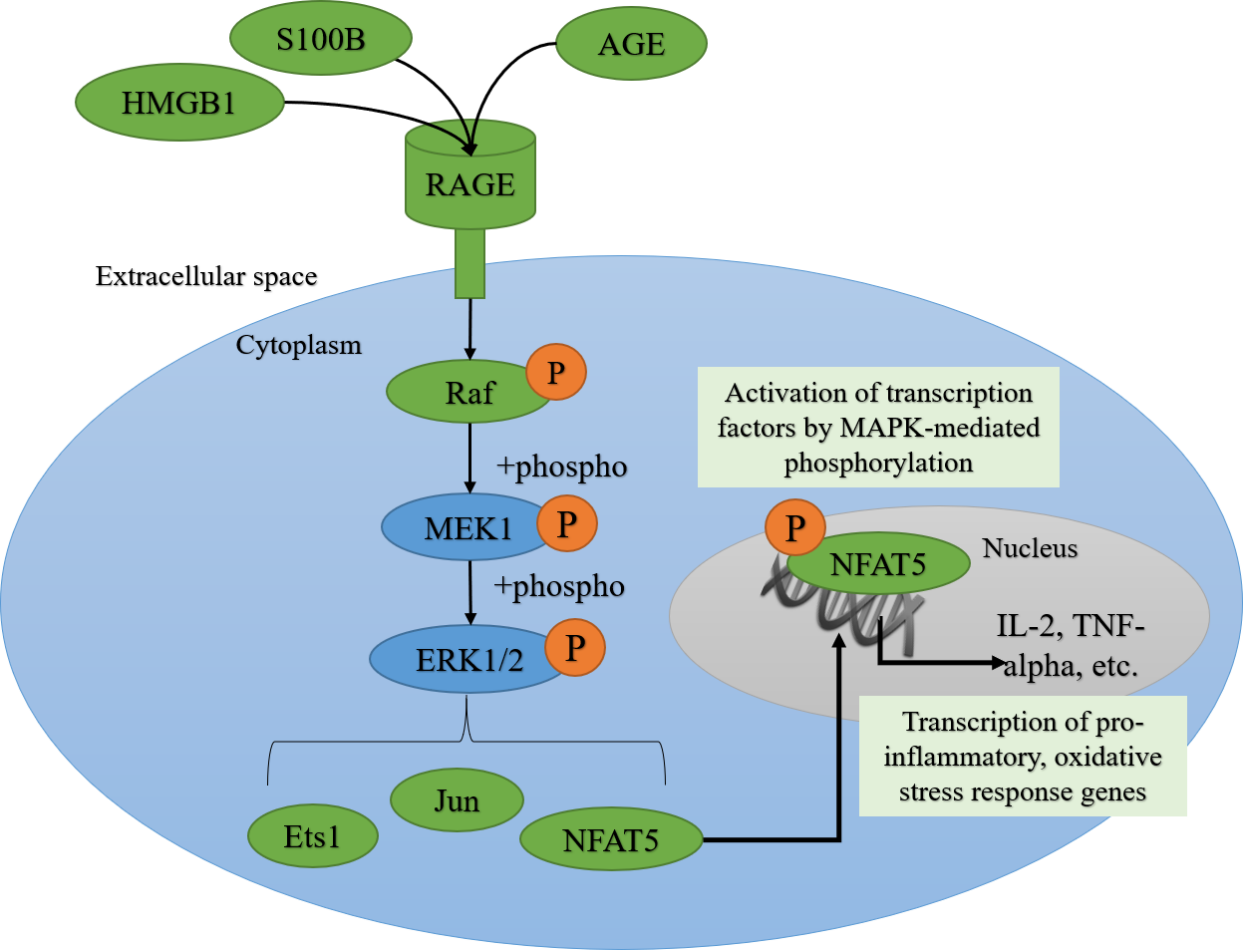
AGE-RAGE signaling can activate ERK1/2 signaling and downstream pro-inflammatory transcription factors. The advanced glycation end-product (AGE)—AGE receptor (RAGE) pathway can be activated by the binding of damage-associated molecular pattern molecules (DAMPs) like S100B (S100 calcium-binding protein B) and HMGB1 (high mobility group box 1 protein) from dying cells, or AGEs, derived from oxidized proteins, lipids, and nucleic acids. RAGE can signal through the MAPK (Mitogen-activated protein kinase) signaling transduction proteins like Raf (MAPK kinase kinase), MEK1 (MAPK kinase), ERK1/2 (Extracellular Signal-Regulated Kinase), leading to the phosphorylation and nuclear translocation of key transcription factors like Ets1, Jun and NFAT5 (Nuclear factor of activated T-cells 5).

In the current study, total protein levels of full length RAGE, HMGB1 and S100B were assessed via Western blotting. RAGE activation via AGEs and DAMPs can induce ERK1/2 signaling (Lander et al., 1997; Reddy et al., 2006) so RT-qPCR was used to evaluate the expression of *rage*, *hmgb1*, one MAPK gene upstream of ERK1/2 (*araf*) and select downstream transcription factors known to be activated by ERK1/2 (*ets1*, *nfat5, jun*) (Fig. 1). The results of this study suggest that RAGE signaling is activated in BAT and WAT upon arousal from hibernation, but CML-AGE, HMGB1 and S100B are probably not responsible for the activation of this pathway.

## Methods

### Animal experiments

Wild-captured 13-lined ground squirrels weighing approximately 150–300 g, were caught and transported by a United States Department of Agriculture-licensed trapper (TLS Research, Bloomingdale, IL, USA) to Dr. J.M. Hallenback’s laboratory at the Animal Hibernation Facility, National Institute of Neurological Disorders and Stroke (NIH, Bethesda, MD, USA), where the hibernation experiments were performed. NINDS animal care and use committee (ACUC) animal housing and experimental procedures were followed (protocol number ASP 1223-05). Each ground squirrel was anesthetized with 5% isofluorane and fitted with a sensor chip (IPTT-300; Bio Medic Data Systems, Seaford, DE, USA) injected under the skin. Each squirrel was housed individually in a shoebox cage at 21 °C. Animals were fed a standard rodent diet and water *ad libitum* until they gained sufficient lipid stores to enter hibernation. To enable a natural transition into torpor, animals were transferred to an environmental chamber at ∼5 °C in constant darkness. Sampling points throughout the torpor-arousal cycle were chosen based on body temperature (*T*_*b*_), time, and respiration rates. The *T*_*b*_ of euthermic in the cold room (EC) animals was stable at ∼37 °C in the 5 °C cold room, and although they could enter torpor, they had not done so for at least 3 days. Entrance (EN) animals were in the entrance phase of the hibernation bout (*T*_*b*_ = 18–31 °C) characterized by decreasing *T*_*b*_. Early torpid (ET) animals had a stable *T*_*b*_ of 5–8 °C for one day. Late torpid (LT) animals had *T*_*b*_ values of 5–8 °C and were continuously in deep torpor for at least 5 days. Early aroused (EA) animals had an increased respiratory rate of more than 60 breaths/min and a rising *T*_*b*_ of 9–12 °C. Interbout aroused (IA) animals were sampled after a multi-day torpor and had a core body temperature returned to ∼37 °C for approximately 18 h. All ground squirrels had been through a series of torpor-arousal bouts prior to sampling. Each animal was anesthetized with isoflurane and decapitated within 2 min from its removal from the hibernation chamber, as per McMullen & Hallenbeck (2010). Tissue samples were shipped to Carleton University on dry ice and were stored at −80 °C until use.

### Total protein extraction

Frozen brown adipose tissue (BAT) and white adipose tissue (WAT) samples were weighed for immunoblotting (∼70 mg of 4 biological replicates from EC, EN, ET, LT, EA and IA) and carboxymethyl-lysine-ELISAs (∼50 mg of 4 biological replicates from EC and LT ground squirrels). Frozen samples were weighed and crushed into small pieces under liquid nitrogen. Five to seven piston strokes of a glass Dounce pestle homogenized the tissues in ice-cold Cell Signaling Lysis Buffer (#43-040; EMD Millipore, Burlington, MA, USA), added in a 1:3 w:v ratio and supplemented with 1 mM sodium orthovanadate, 10mM sodium fluoride, 10 mM *β*-glycerophosphate, and 10 µL/mL Protease Inhibitor cocktail (#PIC001.1; BioShop, Burlington, Canada). Each sample was placed on ice and gently vortexed every 10 min for 30 min before centrifugation at 13,500×g for 20 min at 4 °C. The supernatant containing soluble proteins was collected for each sample and total protein concentration was determined for immunoblotting and ELISA samples using the Bio-Rad method (Cat#500-0005; Bio-Rad, Hercules, CA, USA). Appropriate amounts of homogenization buffer were added to the samples intended for immunoblotting such that they were all standardized to 10 µg/µL. Then, 2X sodium dodecyl sulfate (SDS) loading buffer (100 mM Tris-base adjusted to pH 6.8, 4% w:v SDS, 20% v:v glycerol, 0.2% w:v bromophenol blue, 10% v:v 2-mercaptoethanol) was added to each sample in a 1:1 v:v ratio and the samples were mixed and boiled. The final 5 µg/µL protein samples were stored at −40 °C until use. The samples intended for ELISAs were standardized to 1.4 µg/µL (WAT) and 20 µg/µL (BAT) by adding appropriate amounts of phosphate buffered saline (PBS) containing 0.1% v:v bovine serum albumin (BSA; Sigma, #A4503), as per the commercial ELISA manufacturer’s instructions.

### Western blotting

Equal amounts (25 µg) of prepared protein homogenate and 4–5uL of 10.5–175 kDa PiNK Plus pre-stained protein ladder (#PM005-0500; FroggaBio, Toronto, Canada) were loaded onto 10–15% SDS-PAGE gels, electrophoresed for 80–100 min at 180 V using the BioRad Mini Protean III system in Tris-glycine running buffer (0.25 M Tris-base pH 8.0, 2.45 M glycine, 0.035 M SDS). Proteins were wet-transferred onto polyvinylidene fluoride (PVDF), either at 160 mA for 90 min (RAGE) or at 30 V for 45 min (S100B and HMGB1) in Tris-glycine transfer buffer (25 mM Tris pH 8.5, 192 mM glycine and 10% v:v methanol). Blots were blocked with 2.5–10.0% w:v milk in Tris-buffered saline with Tween-20 (TBST; 50 mM Tris–HCl, 150 mM NaCl, 0.05% v:v Tween-20, pH 6.8) for 15–30 min. The blots were incubated overnight (RAGE) or twice overnight (S100B and HMGB1) at 4 °C on a rocker with primary antibodies (diluted 1:1,000 v:v in TBST). RAGE (#GTX23611; GeneTex, Irvine, CA, USA) and S100B (#GTX129573; GeneTex, Irvine, CA, USA) were rabbit polyclonal primary antibodies and the HMGB1 (University of Iowa, #PCRP-HMGB1-3A7) was a mouse monoclonal primary antibody. Following primary antibody removal, the membranes were incubated for 15 min with an HRP-linked anti-rabbit IgG secondary antibody (RAGE and S100B) or an HRP-linked anti-mouse IgG secondary antibody (HMGB1), diluted 1:8,000 v:v with TBST. Enhanced chemiluminescence (ECL) reagents were used to visualize the protein bands. The amount of protein in each lane was reassessed using Coomassie Blue staining (0.25% w:v Coomassie brilliant blue, 7.5% v:v acetic acid, 50% methanol) of the PVDF membranes.

### Carboxymethyl lysine-advanced glycation end product (CML-AGE) ELISA

CML-AGE was assessed using a protein ELISA because it is the most abundant subtype of AGE, it is formed from the oxidation of protein and lipid precursors, it is a bona fide RAGE ligand, and it induces strong inflammatory signaling through RAGE. The OxiSelect N ε-(carboxymethyl) lysine (CML) competitive ELISA (#STA-816; Cell Biolabs Inc., San Diego, CA, USA) was performed as directed by the manufacturer. All materials listed were included with the kit. Briefly, 100 µL 1X CML Conjugate (prepared by diluting the 1000X CML Conjugate with 1X CML Diluent, prepared in 1X PBS buffer) incubated in CML conjugate-coated wells overnight at 4 °C. Wells of the were washed twice with 1X PBS and then wells were blocked with 200 µL Assay Diluent for 1 h at room temperature while rocking on an orbital shaker. The CML-BSA standard curve was prepared from 1 mg/mL CML-BSA Standard, diluted with Assay Diluent to concentrations between 0–12.5 µg/mL. Then, 50 µL of the standards or WAT (1.4 µg/µL) or BAT (20 µg/µL) samples (EC vs. LT) were added to the CML-Conjugate coated wells in duplicate and incubated for 10 min at room temperature on an orbital shaker. Following a 1-hour (room temperature, shaking) incubation with 50 µL of anti-CML primary antibody (diluted 1:1000 v:v in Assay Diluent), the wells were washed with 1X Wash Buffer. Then, 100 µL of the secondary antibody-HRP Conjugate (diluted 1:1,000 v:v in Assay Diluent) was incubated for 1 h (room temperature, shaking). The wells were washed with 1X Wash Buffer and then 100 µL room temperature Substrate Solution was added to each well and incubated for 3 min. 100 µL of Stop Solution was added to stop the enzyme reaction and the plate was read at 450 nm using a BioTek PowerWave HT spectrophotometer.

### RNA extraction, cDNA synthesis and quantitative RT-PCR

RNA extractions were performed using the TRIzol (Invitrogen, #15596026) method as directed by the manufacturer and as previously described (Logan et al., 2016). Briefly, approximately 100 mg of BAT and WAT was weighed and homogenized in 1 mL of TRIzol using a Polytron PT1200 homogenizer. Four biological replicates were made for each tissue from independent animals for each of the 6 experimental time points, except three WAT biological replicates were prepared for EN and EA due to tissue availability of these time points. RNA purity was assessed using the ratio of absorbances 260/280 nm and RNA integrity was assessed by visualizing 18S and 26S ribosomal bands on a 1% agarose gel with SybrGreen staining. RNA samples were normalized in a total volume of 10 µL DEPC-autoclaved ddH_2_O such that each biological replicate contained 4 µg (brown adipose tissue) or 1.14 µg (white adipose tissue) of RNA. The normalized RNA samples were incubated with 1 µL of Oligo-dT (200 ng/µL 5′-TTTTTTTTTTTTTTTTTTTTTTV-3′; where V = A, G, or C; Sigma Genosys) and placed in a thermocycler at 65 °C for 5 min, then chilled on ice for 5 min. Reverse transcription was performed with 4 µL of 5X first-strand buffer (Invitrogen, #18057018), 2 µL of 0.1 M DTT (Invitrogen, #D1532), 1 µL of 10 mM dNTPs (#NUC001; BioShop, Burlington, Canada), and 1 µL of MMLV Reverse transcriptase (Invitrogen, # 28025013). Following a 45 min incubation in the thermocycler at 42 °C, RT-qPCR was performed as described previously (Pellissier et al., 2006), using a BioRad CFX Connect apparatus. Forward and reverse primers used for RT-qPCR were as follows: *rage,* 5′-AAGCGGGAGAAGCAGAAAGT-3′, 5′-GTGCCAGCTAAGAGTTCCCT-3′; *hmgb1,* 5′-AGAGCGGAGAGAGTGAGGAG-3′, 5′-TGACATTTTGCCTCTCGGCT-3′; *araf*, 5′-TGTACCTGCCCAACAAGCAA-3′, 5′-GTAGACCACGCAGCAATCCT-3′; *jun*, 5′-GAGAGATTGTCGGGGCTGAG-3′, 5′-CCCTTGGCTTTAGTCCTCGG-3′; *ets1,* 5′-AAGCTCTAAGGTGGTCTCAGT-3′, 5′-GCTTCACTTTTCCAATGGGGTC-3′; *nfat5*, 5′-AGCATCCATCAACCCCGAAG-3′, 5′-CCAATCCACACCCCTCATCC-3′; *acta1*, 5′-AGAACAGCAGGTGTAGTCACG-3′, 5′- AGCCATTGTCACACACGAGG-3′; and *tbp*, 5′- AGAGTGTGCTGGGAATGCTC-3′, 5′-CAGGCTGCTGTTCTGATCCA-3′. Finally, isolated PCR products were sequenced (BioBasic, Markham, ON) and BLASTn was used to confirm the identity of the amplified genes.

### Statistical analyses

Western blot bands were imaged using the Chemi-Genius Bioimaging system (Syngene, Frederick, MD) and were quantified using GeneTools software. Background was accounted for. PVDF membranes were stained using Coomassie Blue staining (0.25% w:v Coomassie brilliant blue, 7.5% v:v acetic acid, 50% methanol) to visualize the total amount of protein in each lane. Chemiluminescent band density in each lane was standardized against the summed intensity of a group of Coomassie stained protein bands in the same lane (Eaton et al., 2013). Data are expressed as means ± SEM, *n* = 4 independent samples from different animals for all experiments except an *n* = 3 was used for WAT EN and EA for RT-qPCR experiments, and an *n* = 3 was used for BAT LT in the CML-AGE ELISA experiment, due to limited sample availability. CML-AGE protein concentrations were determined using the CML-BSA standard curve (a graph of mean OD 450 nm ± standard deviation collected for known concentration values). A Student’s *t*-test was used to evaluate the results of the CML-ELISA. Statistical analysis of RT-qPCR experiments involved converting raw Ct values obtained from each PCR run to a linear form using 2-ˆCt calculations (ΔCt). Then the ΔCt values for each gene of interest were normalized against the reference genes (*acta1* for WAT and *tbp* for BAT) (ΔΔCt). For time-course experiments (Western blotting and RT-qPCR), any differences between control and other torpor-arousal time points were analyzed using a one-way ANOVA with Tukey’s post-hoc test. Statistical analyses were performed on SigmaPlot software and considered statistically significant the tests yielded a result of *p* < 0.05.

## Results

### Relative changes in protein levels of RAGE and its ligands, S100B and HMGB1, over the torpor arousal cycle in brown and white adipose tissue

**Figure 2:**
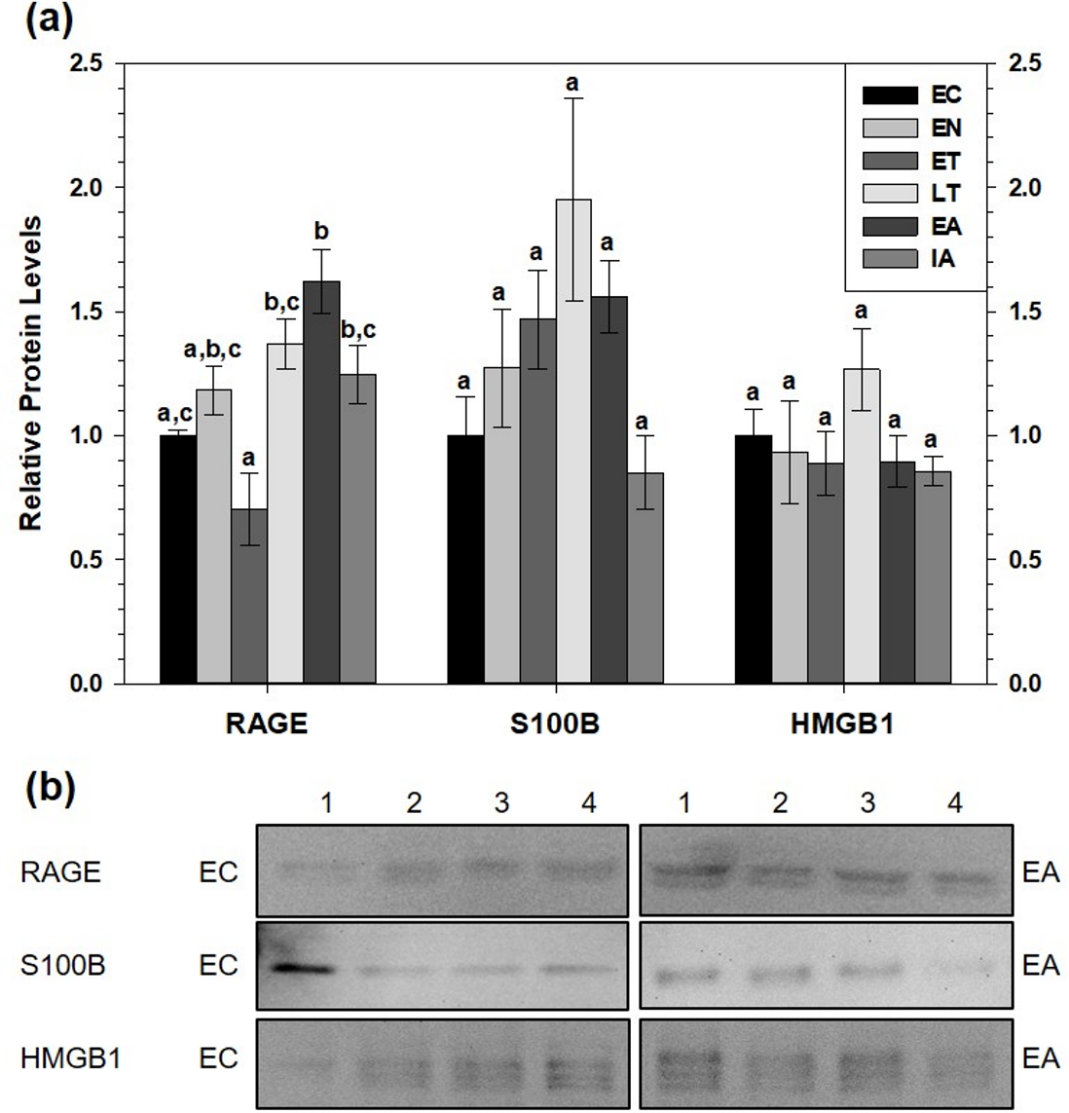
Relative total protein levels of RAGE andligands S100B and HMGB1 in brown adipose tissue (BAT) of 13-lined groundsquirrels. (A) Histogram showing relative mean protein levels of RAGE, S100B, and HMGB1 (± S.E.M., *n* = 4 independent protein isolations from different animals). (B) Representative western blots for certain torpor-arousal time points. A space between representative band images indicates a removal of other lanes from the same blot. Data were analyzed using ANOVA with Tukey’s post-hoc test. Shared letters indicate data that are not significantly different from each other and different letters indicate statistical significant differences between sample points (*p* < 0.05).

Immunoblotting was used to determine relative total protein levels of RAGE, S100B and HMGB1 in brown adipose tissue (BAT) over six time points of the torpor-arousal cycle, to determine if this pro-inflammatory pathway was activated (Fig. 2). Despite no changes in S100B or HMGB1 total protein levels, RAGE total protein levels significantly increased by 1.6-fold during early arousal (EA) with respect the group of euthermic ground squirrels in the cold room (EC) (*p* < 0.05). Furthermore, RAGE levels during late torpor (LT), EA, and interbout arousal (IA) were higher than early torpor (ET) levels (by 2.0-fold, 2.3-fold and 1.8-fold respectively, all *p* < 0.05).

**Figure 3:**
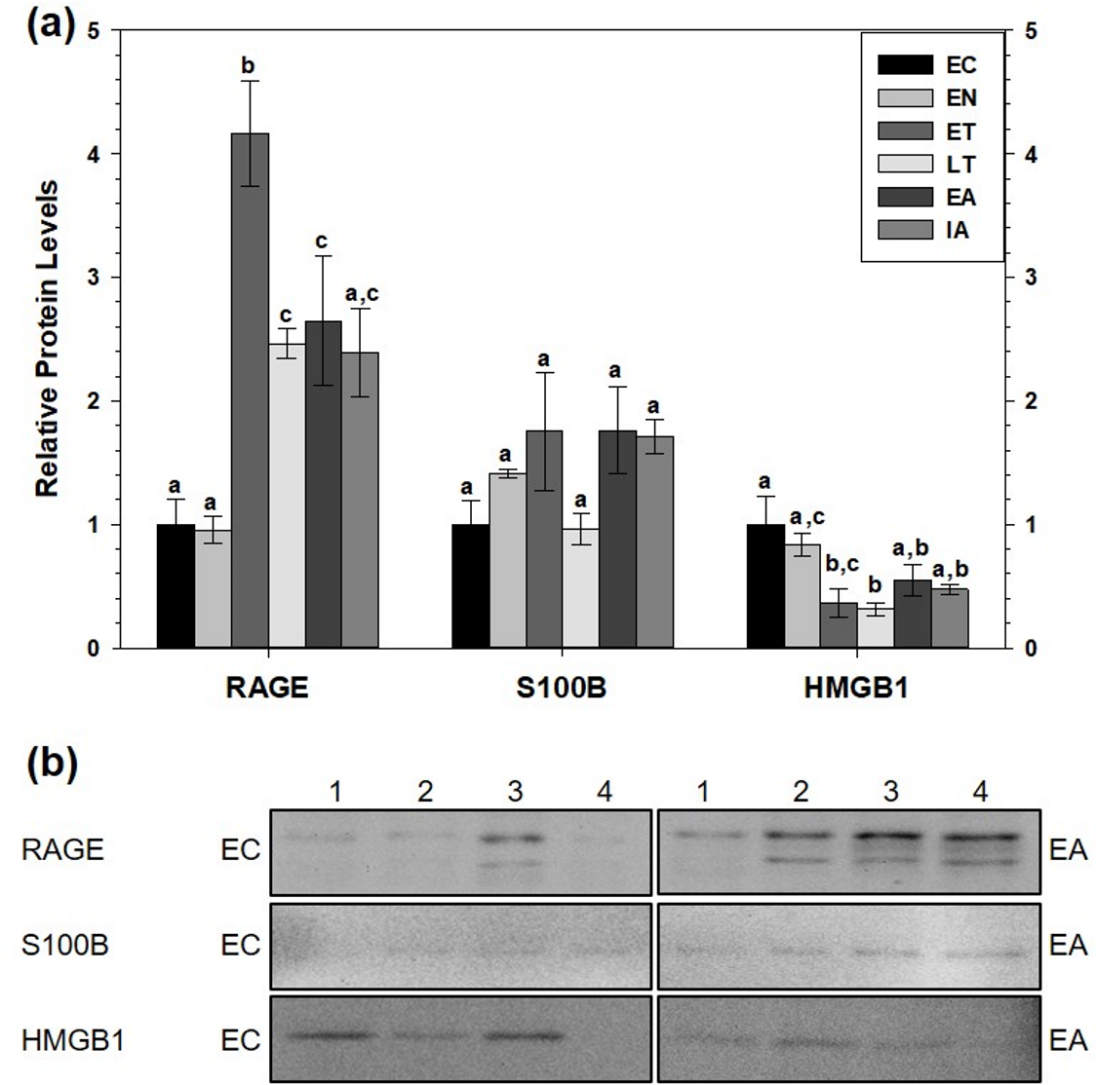
Relative total protein levels of RAGE andligands S100B and HMGB1 in white adipose tissue (WAT) of 13-lined groundsquirrels. (A) Histogram showing relative mean protein levels of RAGE, S100B, and HMGB1 (± S.E.M., *n* = 4 independent protein isolations from different animals). (B) Representative western blots for certain torpor-arousal time points. A space between representative band images indicates a removal of other lanes from the same blot. Data were analyzed using ANOVA with Tukey’s post-hoc test. Shared letters indicate data are not significantly different from each other and different letters indicate statistical significant differences between sample points (*p* < 0.05).

RAGE protein levels also increased in WAT (Fig. 3). Compared to EC, RAGE increased during ET (by 4.2-fold), during LT (by 2.5-fold) and during EA (by 2.6-fold). RAGE levels were significantly higher during ET than at the LT, EA and IA time points. Like BAT, white adipose tissue (WAT) showed no changes in relative S100B protein levels across the torpor-arousal cycle but HMGB1 levels significantly decreased during ET and LT compared to EC to 37% and 31% of the euthermic level, respectively (*p* < 0.05).

### Relative levels of carboxymethyl-lysine advanced glycation end products (CML-AGE) in WAT and BAT, comparing interbout aroused animals to late torpid animals

**Figure 4:**
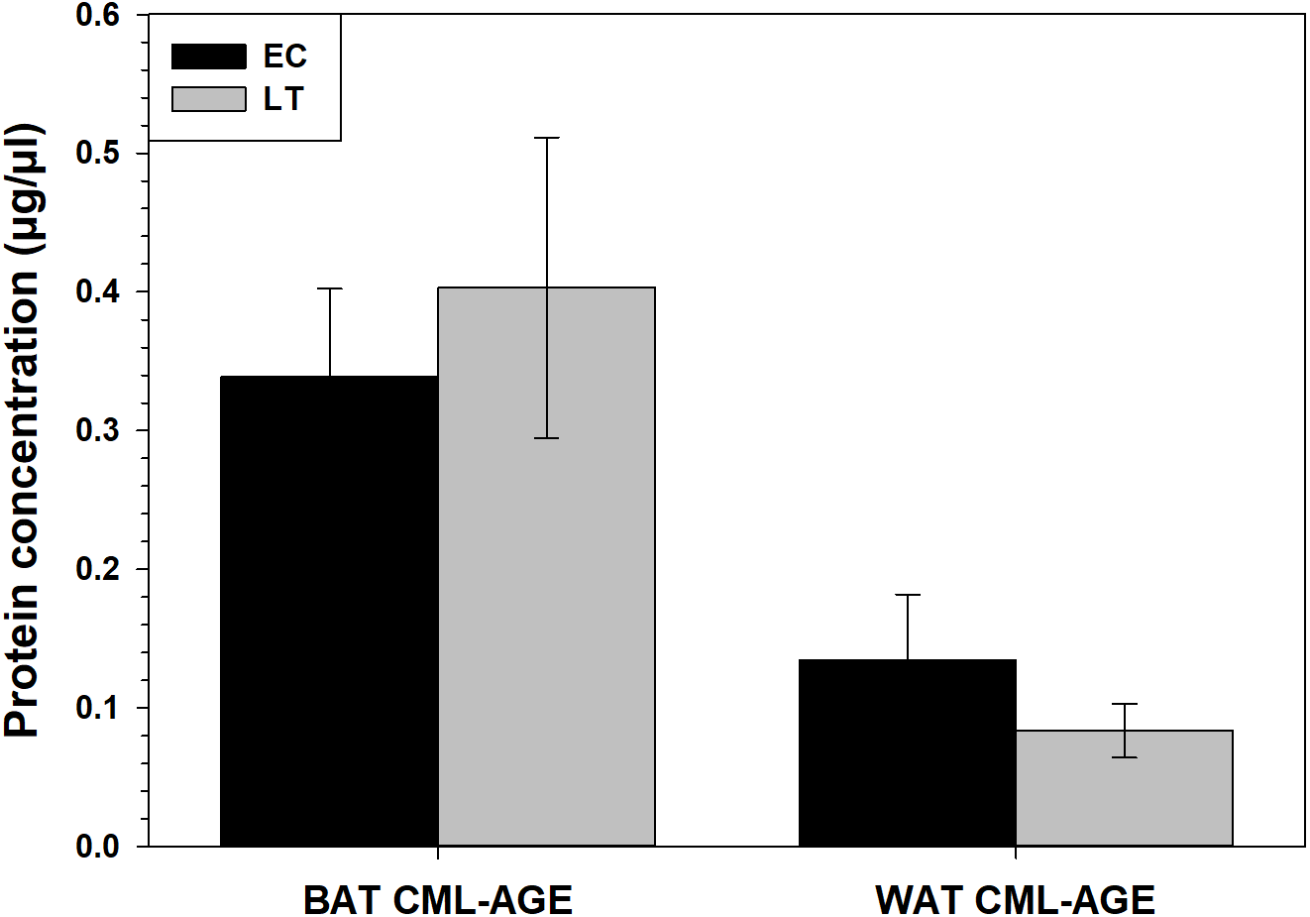
Calculated concentrations of carboxymethyl-lysine (CML) AGE in 13-lined ground squirrel BAT and WAT. Calculated concentrations of carboxymethyl-lysine (CML) AGE in 13-lined ground squirrel BAT and WAT, using a BSA-AGE standard curve (File S1). Values are expressed as mean protein concentrations (± S.E.M., *n* = 4 for all time points except for *n* = 3 for BAT LT). Data were analyzed with a Student’s *t*-test and amount of CML-AGE was deemed not significantly different between LT and EC.

CML-AGE levels did not change in WAT or BAT during torpor, with respect to euthermic 13-lined ground squirrels (Fig. 4). Using the equation of the line (*R*^2^ = 0.9049) obtained from the CML-BSA standard curve, it was determined that CML-AGE concentrations in BAT were 0.33 ±  0.06μg/µL during EC and 0.40 ±  0.11μg/µL during LT. WAT CML-AGE concentrations were much lower with EC levels at 0.13 ± 0.05 and LT values around 0.08 ±  0.02μg/µL.

### Assessment of RAGE, HMGB1 transcript levels and downstream MAPK signaling and transcription factor mRNA levels in brown and white adipose tissue

**Figure 5:**
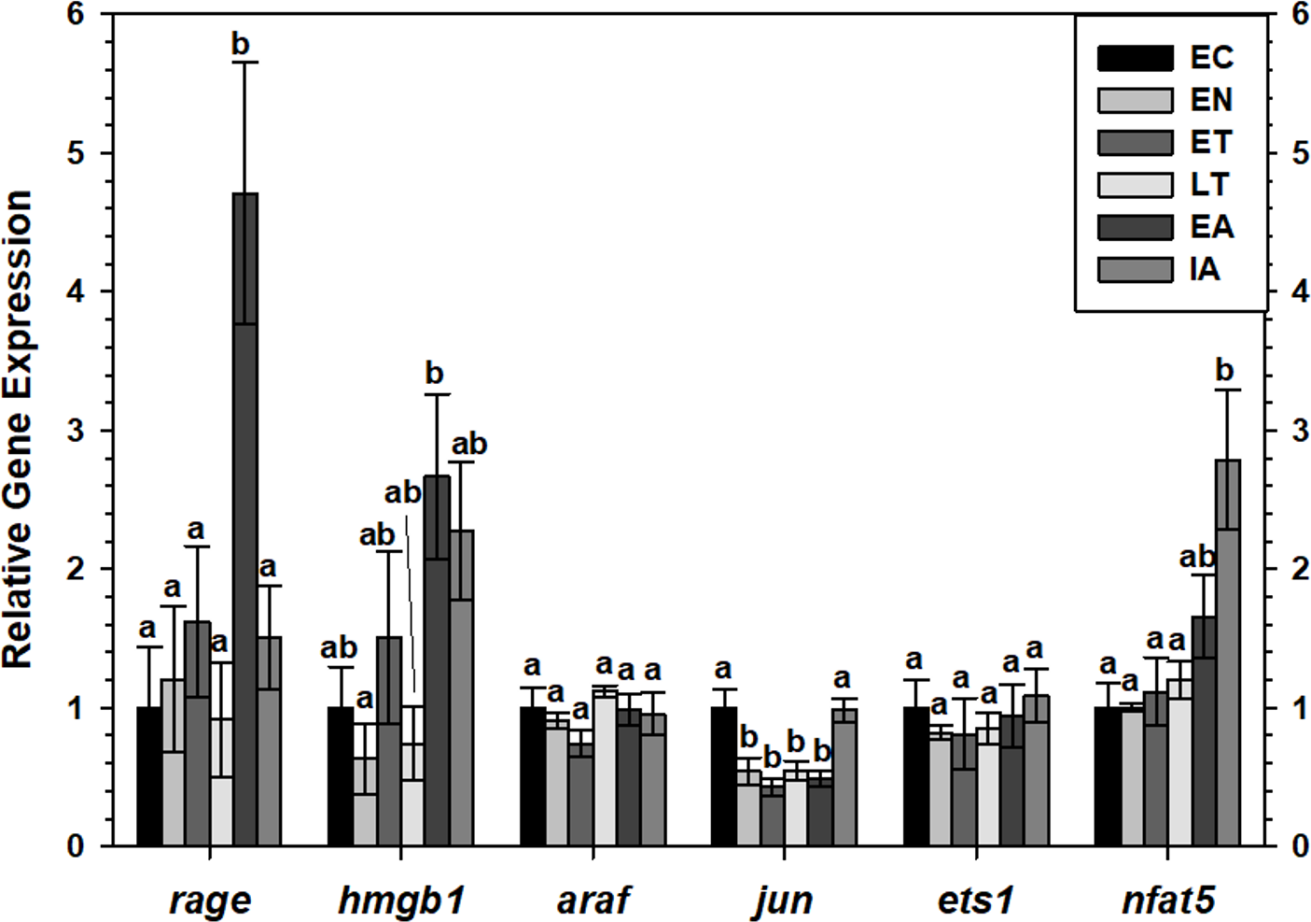
Expression of mRNAs in BAT samples wasevaluated by RT-qPCR for 6 time points over the torpor-arousal cycle. Relative expression of mRNAs was normalized to the expression of TATA-binding protein (*tbp*) mRNA from the same sample. The relative mRNA expression in the torpid animals was normalized to the euthermic control. Data are mean ± SEM (*n* = 4 independent trials from different animals). Data were analyzed using a one-way ANOVA with Tukey’s post-hoc test. Shared letters indicate data that are not significantly different from each other and different letters indicate statistical significant differences between sample points (*p* < 0.05).

**Figure 6:**
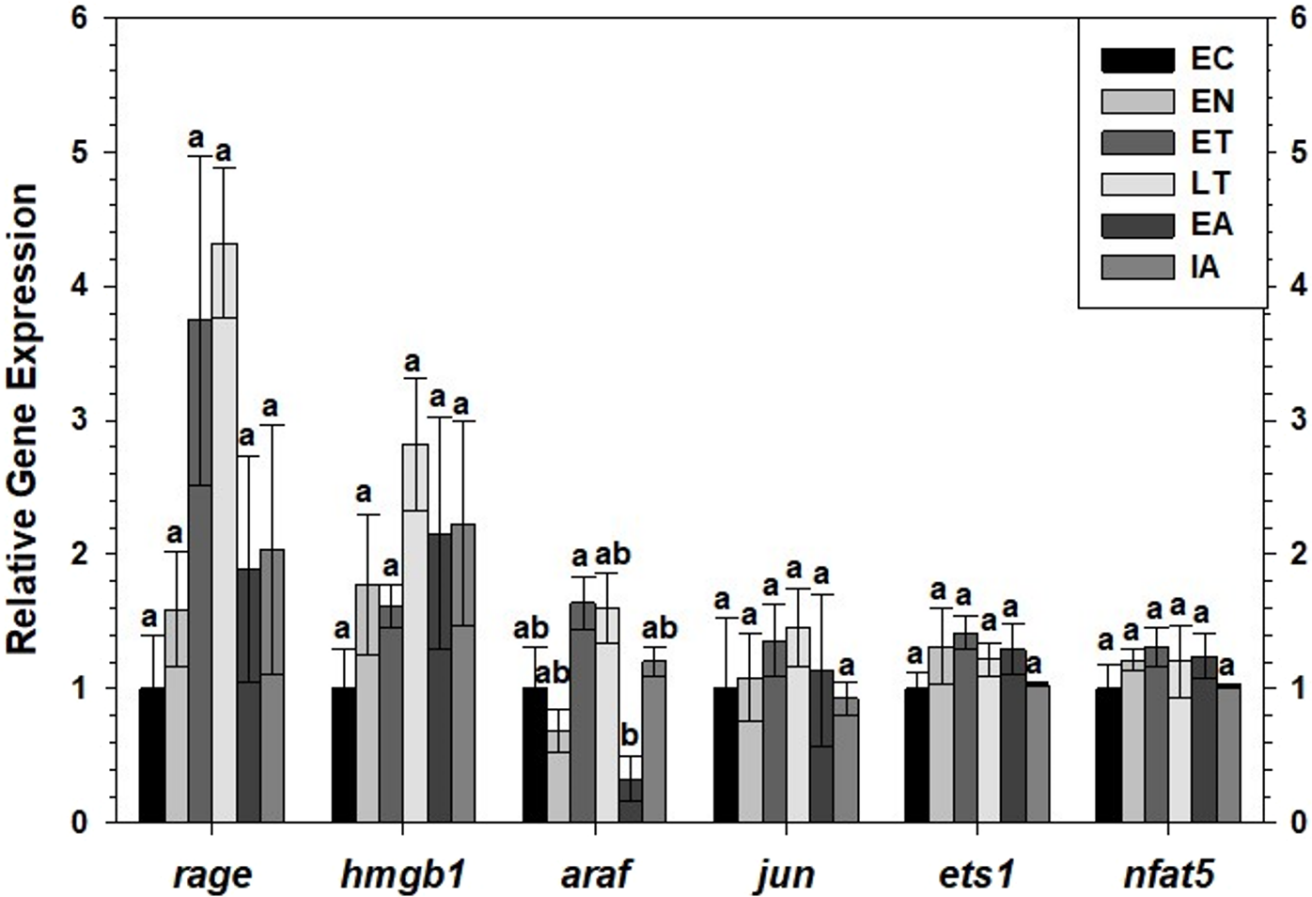
Expression of mRNAs in WAT samples wasevaluated by RT-qPCR for six time points of the torpor-arousal cycle. Relative expression of indicated mRNAs was normalized to the expression of alpha actin (*acta1*) mRNA from the same sample. Samples were made from four independent biological replicates for all time points except *n* = 3 used for EN and EA. Data were analyzed using ANOVA with Tukey’s post-hoc test. Shared letters indicate data that are not significantly different from each other and different letters indicate statistical significant differences between sample points (*p* < 0.05).

Transcript levels of *rage* and *hmgb1* were assessed over the course of the torpor-arousal cycle in BAT (Fig. 5) and WAT (Fig. 6) of 13-lined ground squirrel. BAT *rage* levels remained constant until EA when they increased 4.7-fold (relative to EC) before returning to EC levels during IA. BAT *hmgb1* levels also remained relatively constant but increased during EA relative to EN (to 2.7-fold of EC) but this change was not significant with respect to the control. There was no difference in WAT *rage* and *hmgb1* levels over the torpor-arousal cycle.

RT-qPCR was also used to assess the relative change in gene expression of select MAPK signaling transducers and downstream transcription factors known to be involved in the immune response and oxidative stress response. BAT *jun* levels decreased between EN and EA to 43–55% of the EC level before increasing to euthermic levels during IA (Fig. 5). BAT *nfat5* levels increased significantly during IA to 2.8-fold the EC value, however, *araf* and *ets1* mRNA levels did not change. Similarly, there were virtually no changes in *araf*, *jun*, *ets1*, or *nfat5* mRNA levels in WAT, except for a significant decrease in *araf* levels between during EA relative to ET (Fig. 6).

## Discussion

The AGE-RAGE signaling pathway, which is now understood to be a key pathway in inflammatory disease pathogenesis (including obesity and diabetes) and natural aging mechanisms, has never been studied in hibernators to date, despite the continued interest in understanding how hibernators are able to emerge from torpor unharmed after repeated bouts of torpor (which involves changes in oxidative metabolism and ROS production), and lead long lives relative to their size. This study focused on the response of the pro-inflammatory AGE-RAGE signaling pathway in brown adipose tissue (BAT) and white adipose tissue (WAT) from hibernating 13-lined ground squirrels due to the importance the AGE-RAGE pathway in regulating inflammation and oxidative signaling.

Very interestingly, RAGE protein levels increased in both white adipose tissue (WAT) and brown adipose tissue (BAT) of hibernating 13-lined ground squirrels, as determined via immunoblotting. Consistent between the two tissues, RAGE protein levels were elevated during early arousal (EA), suggesting that RAGE may be involved in the response to rapid rewarming and oxidative stress brought on by more rapid breathing (heightened oxygen intake), or increased oxidative metabolism. Brown adipose cells are packed with mitochondria (relative to other tissues) and are able to generate heat via non-shivering thermogenesis during arousals, which involves heightened electron transport chain activity, and this can lead to increased ROS abundance (especially H_2_O_2_ and superoxide [O_2_]^•−^) (Murphy, 2009). BAT RAGE mRNA levels increased during EA as well, suggesting that the arousing ground squirrel may actively transcribe RAGE to sense and respond to oxidative stress. Elevated RAGE protein and mRNA levels during EA are consistent with a few other studies that show a suppression of the immune response during torpor but a resumption of immunity during arousal. For instance, several hibernating species (brown bears, hedgehogs, and hamsters) showed decreased circulating leukocyte levels during torpor but they returned to pre-torpor levels upon arousal (Sahdo et al., 2013). Reduced immune response in torpid animals is also shown by a decrease in antibody production during torpor, decreased rejection of skin allografts, and no immune response to lipopolysaccharide injection (Bouma et al., 2013). Furthermore, the intestines from torpid gray mouse lemurs had decreased levels of pro-inflammatory cytokines but an increased antioxidant capacity (Tessier et al., 2015), while the intestines of interbout aroused (IA) ground squirrels did not get inflamed despite higher levels of pro-inflammatory (interferon γ and TNF-α) and anti-inflammatory (IL-4 and IL-10) signaling proteins during arousal (Kurtz & Carey, 2007). WAT RAGE protein levels increased the most during ET (by over 4-fold the EC level) and remained high during LT and EA, suggesting that pro-inflammatory signaling could be important in WAT during torpor, despite evidence of low inflammatory responses during torpor in other hibernator tissues. Indeed, acute inflammatory responses are generally protective, as they attract anti-inflammatory, immune, and antioxidative agents to the site of inflammation. Based on unchanging WAT rage transcript levels, WAT RAGE may be translated from stored mRNA pools in stress granules rather than actively transcribed over the torpor-arousal cycle. Together, these data suggest RAGE is regulated in a tissue-specific manner, where dormant ground squirrels increase RAGE activity throughout torpor and arousal in preparation for threats to the immune system or elevated oxidative stress upon rewarming.

DAMP proteins HMGB1 and S100B were identified as potential targets for assessing pro-inflammatory signaling in hibernating ground squirrels because both are highly expressed in adipose tissues, and are upregulated in diet-induced obesity mice models and following high fat diets in mice (Buckman et al., 2014; Song et al., 2014; Ghosh et al., 2016). By contrast, most other S100 proteins including S100A8, A9, or A12 are expressed by immune cells (granulocytes, keratinocytes) or epithelial cells (Foell et al., 2007; Buckman et al., 2014). S100B and HMGB1 protein levels remained relatively constant over the torpor-arousal cycle in 13-lined ground squirrel WAT and BAT, except that WAT HMGB1 protein levels decreased during ET and LT. Relatively little change in the levels of these DAMPs could suggest that there are few damaged cells over the course of a torpor-bout and they likely do not play a role in RAGE-mediated signaling. However, low (nano-molar range) levels of S100B are often protective since they bind RAGE to induce adaptive functions like neurite outgrowth in the brain or cell migration. High concentrations of S100B (micro-molar range) produce ROS and induce apoptosis (Sparvero et al., 2009). Thus, constant S100B levels could indicate that ground squirrel WAT and BAT are protected from injury during metabolic suppression and upon arousal. *Hmgb1* transcript levels did not change in WAT but BAT *hmgb1* increased during EA with respect to EN. Hmgb1 mRNA levels were shown to increase following thermal injury in rats (Fang et al., 2002), so this slight increase in BAT *hmgb1* levels during arousal could suggest *hmgb1* is more expressed during heat stress as ground squirrel body temperature rises from ∼5 °C to euthermic levels. Furthermore, elevated *hmgb1* levels from adipose correlate with pro-inflammatory marker levels and metabolic syndrome or tissue damage (Jialal et al., 2015; Qiu et al., 2016), suggesting that ground squirrels may upregulate *hmgb1* during EA in response to oxidative stress. Again, these changes are not observed at the protein level, indicating ground squirrels may inhibit HMGB1 protein expression as a means to reduce the pro-inflammatory response. CML-AGE levels did not change in BAT or WAT either, suggesting that ground squirrels likely prevent AGE accumulation before torpor. A previous study done by our lab revealed that 13-lined ground squirrel WAT and BAT adapt to changes in oxidative stress by increasing antioxidant enzyme levels during torpor (but not during arousal) with respect to a euthermic control. Specifically, thioredoxin 1 (TRX1) increased during LT in BAT and WAT, while superoxide dismutases 1 and 2 (SOD1, SOD2) increased in WAT during LT (Rouble, Tessier & Storey, 2014). Increased antioxidant enzyme levels and activity could deplete the pools of ROS that are required to catalyze the non-enzymatic formation of AGEs. Overall, no changes or decreases in DAMPs (S100B and HMGB1) and CML-AGE reverberate the notion that pro-inflammatory and immune responses are repressed during torpor while the antioxidant response is intensified since these three ligands are typically highly abundant and upregulated in pro-inflammatory environments.

**Figure 7:**
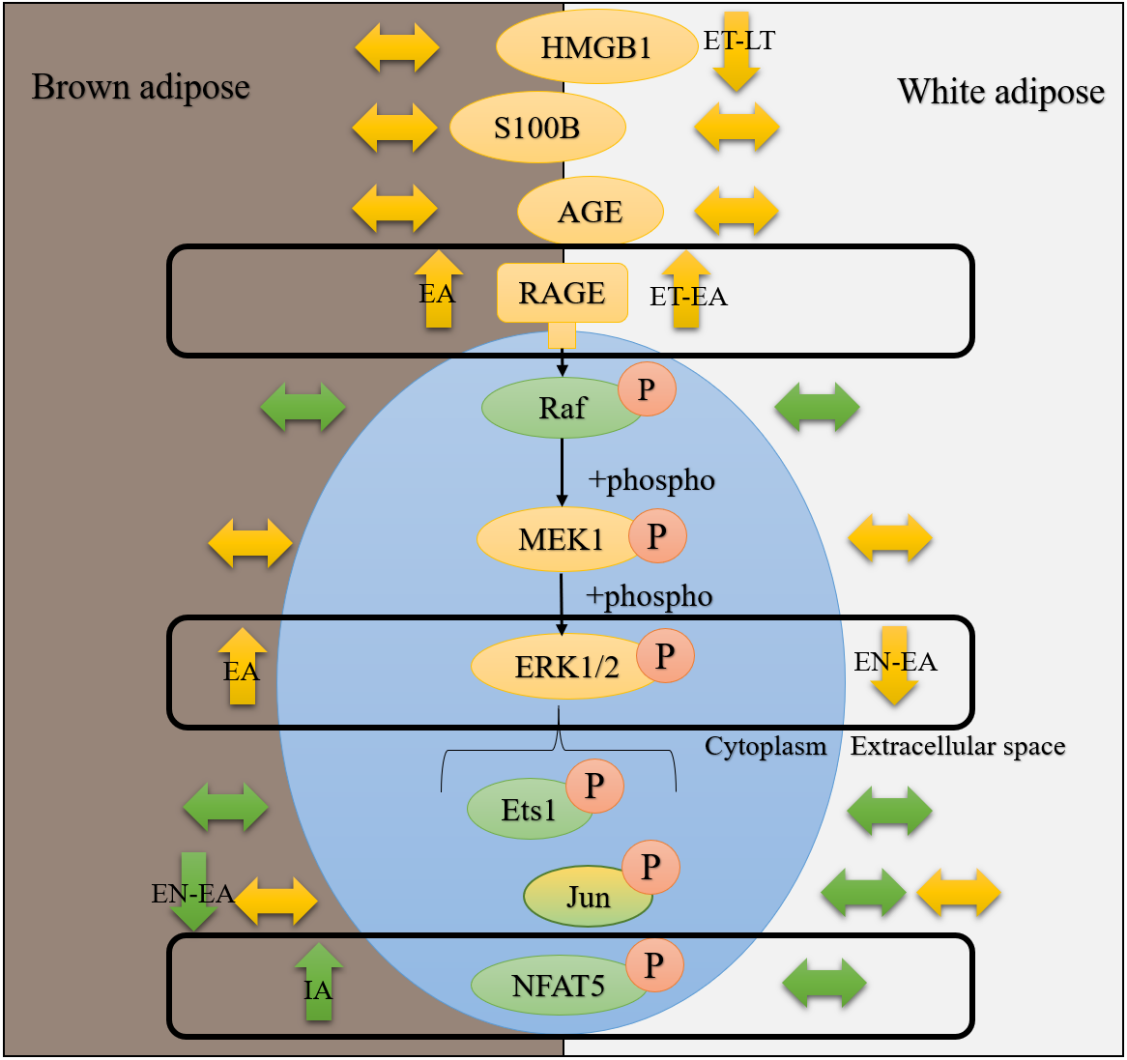
Hibernating 13-lined ground squirrelsdifferentially regulate RAGE and downstream ERK1/2 signaling pathway proteinsin white and brown adipose tissues. Data from the current study and the study by Rouble, Tessier & Storey (2014) were used to generate this figure. Green bubbles represent mRNAs and yellow bubbles denote proteins unless accompanied by an orange “P”, which indicates that data for phosphorylation of this protein is being considered. Arrows pointing up, down, or sideways denote increases, decreases, or no change (respectively) in mRNA, protein, or phosphorylation levels. Time course time points where the changes are observed are indicated in black text on top of the arrows describing the direction of the change.

To assess if AGE-RAGE could induce pro-inflammatory and immune responses at any time point of the torpor-arousal cycle in ground squirrels, RT-qPCR was used to analyze the relative transcript abundance of key MAPK signaling proteins. There is a breadth of literature that indicates that RAGE ligands (AGEs and DAMPs) signal through RAGE to activate MAPKs (specifically, the ERK1/2, JNK, and p38 pathways) (Lander et al., 1997; Qiu et al., 2016) and this leads to increases in gene expression by phosphorylated pro-inflammatory transcription factors (e.g., NFATs, AP1 subunit c-jun, Ets, etc.) (Pulverer et al., 1991; MacDonald & Storey, 2005a; Lu & Xu, 2006; Tsai et al., 2007; Wang et al., 2014) and the activation of transcription activators like p90 RSK S6 kinase (Cowan & Storey, 2003). Furthermore, studies on various ground squirrel tissues indicate that MAPKs are differentially regulated over the torpor-arousal cycle (MacDonald & Storey, 2005b; Rouble, Tessier & Storey, 2014), making them important to discuss in the context of RAGE activation. In ground squirrel WAT, there were no changes upstream of ERK1/2 kinase (i.e., *araf* transcript levels) or in the expression of any transcription factors downstream of ERK1/2 (i.e., *jun*, *ets1,* and *nfat5*). These results are consistent with decreased ERK1/2 phosphorylation during EN, ET and LT, previously described in 13-lined ground squirrel WAT (Rouble, Tessier & Storey, 2014). Overall, the results suggest that RAGE signaling may be active in ground squirrel WAT but it is not inducing downstream MAPK signaling (Fig. 7). Instead, RAGE may induce pro-inflammatory signaling through the NF-κB signaling pathway, since RAGE signaling can increase the *de novo* synthesis of NF-κB (Sparvero et al., 2009), but this has yet to be confirmed in hibernator WAT. NF-κB has been shown to increase in hibernator intestine during torpor, relative to a summer active control, but was undetectable in brown adipose (Carey, Frank & Seifert, 2000). There is also evidence that NF-κB subunits (p50/p65) and the kinase (IKK) that activates NF-κB by inhibiting NF-κB inhibitor (IκB) increase during arousal in 13-lined ground squirrel muscle, while IκB is more phosphorylated during this time, leading to IκB degradation (Allan & Storey, 2012). Thus, RAGE may regulate inflammation through NF-κB in WAT as opposed to MAPK signaling.

Consistent with increased BAT RAGE during arousal, this study showed an increase in BAT *nfat5* transcript expression during arousal. These results also make sense with previous reports of increased BAT ERK1/2 phosphorylation during EA, assessed in *I. tridecemlineatus* (Rouble, Tessier & Storey, 2014). By contrast, BAT *jun* transcript levels decreased between EN and EA, suggesting that this transcription factor is not involved in pro-inflammatory signaling during torpor or arousal. This result is consistent with decreased phosphorylated JNK levels (a kinase that phosphorylates the protein c-jun), observed in BAT and WAT from 13-lined ground squirrels (Rouble, Tessier & Storey, 2014), further emphasizing that this transcription factor is an unlikely mediator of pro-inflammatory signaling in hibernator adipose. Future studies should be directed towards quantifying the relative changes in total and phosphorylated NFAT5 levels to determine if the change in transcript levels matches those at the protein level. Together with the data from the Rouble, Tessier & Storey (2014) study, there is promise that the AGE-RAGE pathway may be involved in the increase in MAPK signaling observed upon arousal in BAT.

## Conclusions

The results of the first-ever AGE-RAGE study in a hibernator have shown that the AGE receptor, RAGE, is more abundant during torpor and arousal in WAT, and during arousal in BAT. RAGE is regulated at the level of transcription in BAT but WAT RAGE protein synthesis could be the result of mobilized mRNA stores. Interestingly, neither DAMPs (S100B and HMGB1) nor CML-AGE are upregulated, suggesting that they are not likely involved in invoking an inflammatory response at any point of the torpor-arousal cycle. Although CML-AGE is usually the most abundant and most potent inducer of RAGE signaling, there are countless other AGE species that could bind RAGE during torpor to increase its upregulation. RT-qPCR analysis implicate that MAPK signaling and downstream transcription factors Jun, NFAT5 or Ets1 were not upregulated over the torpor-arousal cycle in WAT, suggesting that these transcription factors are likely not involved in the pro-inflammatory, immune or oxidative stress responses in hibernator WAT during hibernation. However, NFAT5 transcript levels increased during arousal in brown adipose, which correlated with BAT RAGE levels and previous studies showing increases in phosphorylated ERK1/2 levels during arousal in BAT. Future studies should include the analysis of the key RAGE-induced downstream transcription factor, NF-κB, in ground squirrel adipose over the course of the torpor-arousal cycle.

##  Supplemental Information

10.7717/peerj.4911/supp-1 File S1 CML-BSA competitive ELISA standard curve using optical density at 450 nm Concentrations of carboxymethyl-lysine (CML)-bovine serum albumin (BSA) ranging from 0–12.5 µg/mL with absorbances measured spectrophotometrically at 450 nm.

## Additional Information and Declarations

### Data Availability

The following information was supplied regarding data availability: Logan, Samantha (2018): AGE competitive elisa. figshare. Dataset. https://doi.org/10.6084/m9.figshare.5885032.v1
Logan, Samantha (2018): BioRadCFX files for RT-PCR data. figshare. Fileset. https://doi.org/10.6084/m9.figshare.5885026.v1
Logan, Samantha (2018): AGE-RAGE western blots. figshare. Fileset. https://doi.org/10.6084/m9.figshare.5885029.
