# Supplementary figures and images for: Pro-inflammatory AGE-RAGE signaling is activated during arousal from hibernation in ground squirrel adipose
Source: PeerJ. 2018 Jun 4;6:e4911. doi: 10.7717/peerj.4911 (PMC5991297; doi:10.7717/peerj.4911)

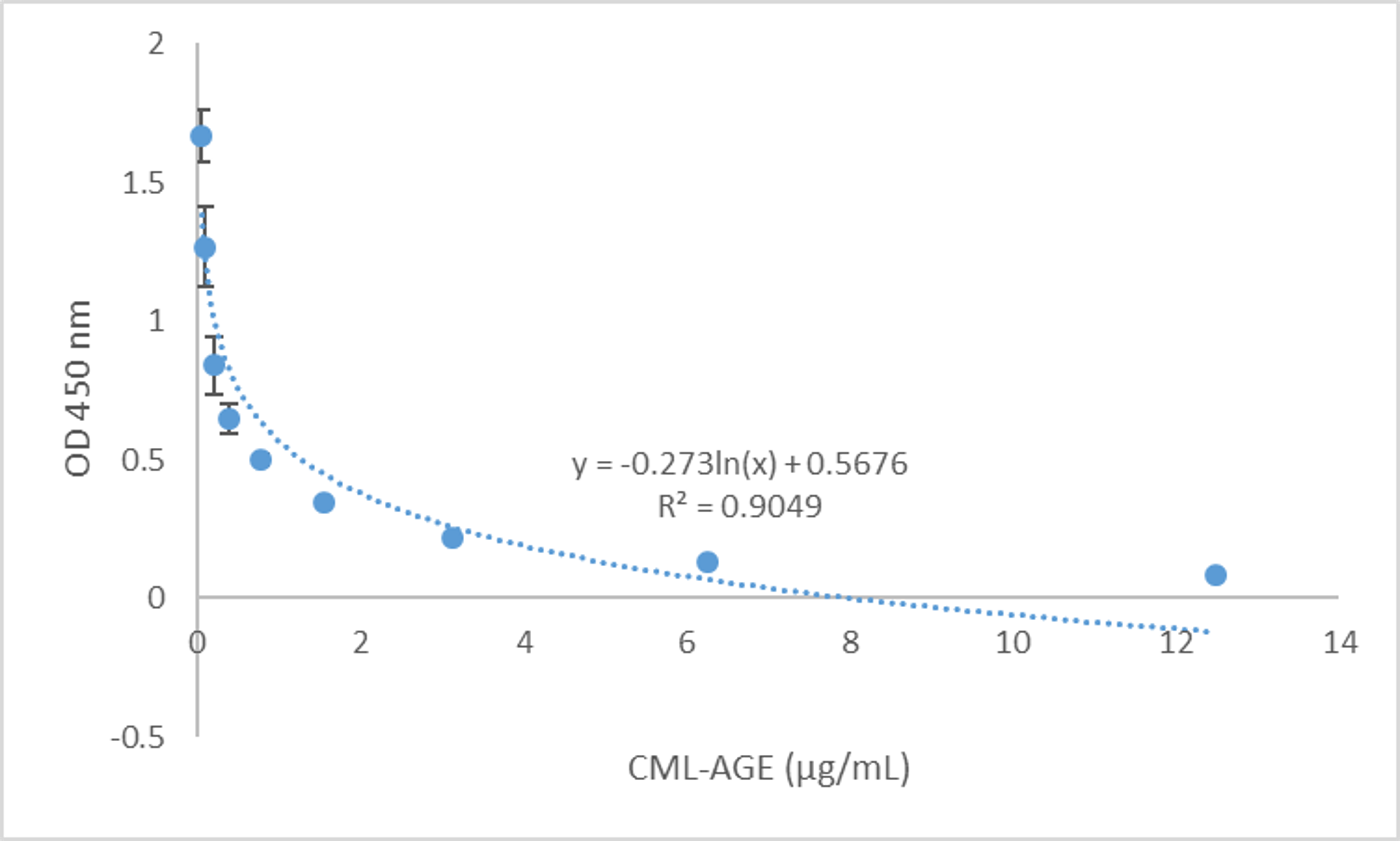

Supplement: File S1 — Concentrations of carboxymethyl-lysine (CML)-bovine serum albumin (BSA) ranging from 0–12.5 µg/mL with absorbances measured spectrophotometrically at 450 nm. [file peerj-06-4911-s001.png]
